# Effect of Pilates exercises on balance and gross motor coordination in children with Down syndrome

**DOI:** 10.1007/s13760-024-02517-w

**Published:** 2024-04-01

**Authors:** Alaa AL-Nemr, Shimaa Reffat

**Affiliations:** Department of Physical Therapy for Pediatrics, Faculty of Physical Therapy, Cairo University, El Tahrir St., Giza, 12613 Egypt

**Keywords:** Balance, Down syndrome, Motor disorders, Rehabilitation, Therapy

## Abstract

**Objective:**

To investigate the effect of Pilates exercises on balance and gross motor coordination in children with Down syndrome (DS).

**Methods:**

Forty children with DS, aged 8 up to 10 years, were randomly divided into two groups; experimental and control groups. A designed physical therapy program was applied for both groups, while the experimental group received an additional Pilates exercise program. Balance and gross motor coordination as primary outcomes and quality of life (QoL) as secondary outcome were assessed using Biodex balance system (BBS), Bruininks Oseretsky of Motor Proficiency (BOT-2), and Pediatric quality of life inventory (PedsQL™) sequentially.

**Results:**

Both experimental (Pilates) and control groups demonstrated significant improvements in dynamic balance, gross motor coordination, and QoL after interventions. However, comparison between groups showed significant improvement in favor of the Pilates group in all measured outcomes (*P* < 0.0001).

**Conclusion:**

Adding Pilates exercises to the designed physical therapy program could provide more significant improvements in balance, gross motor coordination, and QoL in children with DS.

**Trial registration:**

Clinical Trial gov number Identifier: NCT05928949.

## Introduction

The most prevalent genetic developmental disorder in children is Down syndrome (DS) [1]. Children with DS have an extra chromosome 21 and present with many brain dysfunctions. They have a variety of issues, such as cognitive impairments, psychosocial defects, neurological abnormalities, gross motor delays, and health impairments which may affect their quality of life (QoL) [1]. Annually, DS affects 1 in 1000 to 1100 live births worldwide [1]. The capability to sustain one's body in equilibrium is known as balance. This capability is essential for being able to perform movements or motor tasks needed in daily life and can be examined in both static and dynamic conditions [2]. The integration of a variety of biomechanical, motor, and sensory inputs is necessary for balance [2]. Research literature documented that children with DS are characterized by poor balance and gross motor skills when compared to children with typical development [3]. Hypotonia in children with DS indeed negatively affects their balance reactions and motor coordination and relates to proprioceptive feedback deficiencies [3]. The balance deficits represent serious functional limitations resulting in activity and participation restrictions which might affect negatively their QoL [4]. Therefore, balance and body coordination improvement in children with DS represent a key issue [4].

Children with DS can benefit from several exercise interventions to improve their balance, muscle strength, and gross motor coordination. These exercises include balance exercises, muscle strength activities, general physical activities, and combinations of these interventions [4]. Pilates exercises involve movements that activate the balance system's neuromuscular elements, which are crucial for maintaining the stability of the body. They also cause structural and functional changes to the body's balance. These exercises emphasized control of body position and movement [5] and can be used for all age groups by combining coordination, stretching, and strengthening exercises with rhythmic respiratory training [6]. They are based on six fundamental principles that are flexibility, breathing, control, centering, concentration, and precision [5]. Pilates exercises could improve the stability of the spine, muscle strength, and flexibility of the pelvis, and hip joints [7]. Many studies have shown that Pilates exercises could improve balance, and lower limbs strength in children [8–11] however, these studies have not studied children with DS and still, the effect of Pilates exercises in children with DS is unknown. It was hypothesized that there is no effect of Pilates exercises on balance and gross motor coordination in those children.

## Methods

### Design

This research was conducted between Jan 2023 and Aug 2023, with a single-blinded, randomized, controlled design. Two groups of forty children with DS, experimental and control groups, were randomly assigned using opaque sealed envelopes. Twenty children were included in each group. The control group received a designed physical therapy program, each session lasted approximately 90 min, while the experimental group received the same designed physical therapy program administered to control group for 45 min each session in addition to Pilates exercises for another 45 min. Both groups received the above-mentioned exercises 3 sessions/week, for 12 consecutive weeks. The assessment was performed by a blinded outcome assessor twice; at baseline and immediately post-treatment.

## Participants

Children with DS from both sexes were selected according to the following criteria: diagnosed with Trisomy 21 through genetic testing; their ages varied from 8 to 10 years; had a mild or moderate mental disability to adhere to instructions given during assessment; and they were able to stand and walk independently. Exclusion criteria included children with musculoskeletal limitations to exercise such as spinal, hip, knee, or foot deformities; uncontrolled cardiovascular disorder; problems in vision or hearing that hinder exercise performance.

## Intervention

Children in the control group followed a specially designed physical therapy program. Exercises were performed by an experienced pediatric physical therapist. The program included: (1) flexibility exercises for both hamstrings, calves, hip adductors, and flexors; (2) strengthening exercises for abdominal, back, hip abductors, hip extensors, hamstrings, and quadriceps; (3) walking for 15 min on an even surface with a comfortable self-selected pace; (4) postural control exercises including step up and down, standing on one leg with eyes opened then, with eyes closed, tandem standing and tandem walking. Exercises were applied according to children’s tolerance level [10].

Children in the experimental group received Pilates exercises which were applied by an experienced pediatric physical therapist. Before starting the first session, the child was informed of the basic principles of Pilates exercises and their application. Sessions in the first 2 weeks were performed with 5 repetitions for each exercise with two minutes rest period before starting the next exercise, and the number of repetitions increased to 10–15 repetitions in subsequent sessions. Children were asked to perform each exercise slowly and smoothly and to stop the exercise if any pain appeared. Also, modifications of the exercises and the progression of the repetitions were allowed and determined according to the physical specifications of the selected children regarding the limitations imposed by DS. Each session included warm-up and cool-down periods for 10 min and the other 35 min for Pilates exercises. Exercises focused on strengthening muscles stabilizing the trunk, and lower limb muscles, maintaining proper spinal and pelvic alignment, and focusing on respiration rhythm [10, 12]. Details of the selected Pilates exercises are described in Table 1.

**Table 1:** Pilates exercises

Pilates exercises	Starting position	Description
Bridge	Crook lying	Lifting the pelvis off the mat
Hundred	Supine	Lifting the head, followed by lifting both legs 30 degrees with both knees flexed and then gradually with both knees extended
Alternate toe taps	Supine with 90 degrees flexion of both hips and knees	Alternate tapping of toes on the mat
Single leg circles	Supine	Make circles with one raised lower limb in a clockwise direction and then in a counter-clockwise direction
Side to side	Crook lying	While the upper body is maintained in the neutral position, the lower body is twisted to the right and then to the left side
Side kick lying	Side-lying	With the upper leg in an abducted position with an extended knee, the child was asked to move the upper leg in flexion and extension as if sideways kicking
Quadruped	Quadruped	Raising reciprocal arm and leg simultaneously
Spine twist	Kneel standing with both shoulders abducted 90 degrees and clasped hands behind the head	Twisting the spine from side to side
Side leg raise	Standing	Hip abduction and adduction
Standing side splits	Standing	Standing on one leg while the other leg performs side splits by moving a ball far and close to the stance leg
Tandem walking	Standing with toes touching the heel of the front foot	Walking heel to toe in a straight line
Ball wall squat	Standing against a wall while a Swiss ball is in the lumber area	Performing semi-squat

## Outcome measures

### Primary outcomes

Biodex Balance System (BBS) is an objective measure with high reliability that is used in assessing balance [13]. It is utilized to test dynamic balance and stability limits. Eight levels of testing are available where level eight indicates ultimate stability and level 1 indicates the lowest stability [14]. It has a dynamic platform that allows assessing the standing balance [14]. It calculates the overall stability index (OSI), mediolateral stability index (MLSI), and anteroposterior stability index (APSI) [15]. We used the “Dynamic Balance” operation as a method of assessment of the stability Index. Prior to the actual initial assessment; the child made a trial test to be familiarized with the testing procedures. The age, height, and weight of each child were recorded in the system. Instructions to focus on the visual feedback presented on the screen and to maintain the cursor in the middle as much as possible were given. After the test was ended, the stability index results appeared on the screen, with higher scores indicating poor balance.

Bruininks-Oseretsky of Motor Proficiency (BOT-2) is a valid and reliable assessment tool used for assessing the fine and gross motor skills from four to twenty-one years old [16, 17]. It consists of 8 subtests with a total of 53 items arranged in fine and gross motor forms. The gross motor form assesses gross motor coordination. In our study, the gross motor form was selected to be assessed. It assesses two main areas: strength and agility and body coordination [17]. The gross motor record form has four subtests: balance with bilateral coordination subtests form together “body coordination”; while strength with running, speed and agility subtests form together “strength and agility”. The summation of all four subtests scale scores forms the “Gross motor composite scale score”. Each subject was encouraged to do the best performance. Each item was scored first in a raw score, which is converted into a point score which is further converted into a standard score, that takes into account both gender and age [18].

### Secondary outcome

Pediatric quality of life inventory (PedsQL™) is a highly reliable scale with moderate to high criterion validity and sound psychometric properties in children who have developmental and intellectual impairments [19]. The participating children's QoL was evaluated using PedsQL™ generic Core Scale. It consists of four generic core scales; physical, emotional, social, and school functioning. The three summary scores are (1) the total scale score, which includes the results from all subscales; (2) the physical score, which only includes the results from the physical functioning subscale, (3) the psychosocial score, which combines the results from emotional, social, and school functioning subscales [20]. Higher scores indicate greater QOL. Items were scored using a 5-point ordinal scale, which was then converted to a 0–100 scale [20]. The parent proxy version was selected to be used in this study as the children with DS had trouble completing the inventory due to their cognitive impairment.

## Sample size calculation

Using the statistical tool "G*POWER" version 3.1.9.4 (Franz Faul, Universitat Kiel, Germany), the sample size was established before the study using *β* = 0.2, *α* = 0.05, and large effect size. The detected sample size for our study was *n* = 40; with 20 children per group. Forty-six children were recruited for possible dropouts.

## Statistical analysis

The characteristics of subjects including age, height, and weight between groups were analyzed using an independent *t*-test. Sex distribution between groups was analyzed using the chi-square test. Within and between treatment effects of dynamic balance, gross motor coordination, and QoL for the Pilates and control groups were compared using mixed MANOVA. For sequent multiple comparisons, Bonferroni correction was utilized. The significance level for all statistical tests was set at (*P* < 0.05). The statistical analysis was carried out using IBM SPSS, Chicago, IL, USA, version 25 of the statistical software for social studies (SPSS) for Windows.

## Results

This study included forty-six children with Down syndrome. Four children failed to meet the requirements for inclusion and the caregivers of two children refused their participation. Forty children were enrolled in this study. The participants’ characteristics are shown in Table 2. Age, height, weight, and distribution of gender revealed non-significant differences between groups (*P* > 0.05).

**Table 2:** Participants’ basic characteristics in Pilates and control groups

	Pilates group		Control group		*P* value
	Mean ± SD	*N* (%)	Mean ± SD	*N* (%)	
Age (years)	8.86 ± 0. 58		9.16 ± 0.48		0.180^a^
Height (cm)	119.45 ± 3.64		120.05 ± 3.98		0.621^a^
Weight (kg)	22.80 ± 2.33		23.15 ± 2.43		0.645^a^
Boys/Girls		8/12 (40%/60%)		6/14 (30%/70%)	0.507^b^

*SD* standard deviation, *N* number, *%* percentage

^a^Independent *t*-test

^b^Chi-square test

### Treatment effect on stability indices, BOT-2, and PedsQL

Treatment and time showed significant interaction; *F* (9,30) = 116.3, *P* < 0.0001, *η*^2^ = 0.972. The treatment had a significant main effect; *F* (9,30) = 72.8, *P* < 0.0001, *η*^2^ = 0.956. Also, the time showed a significant main effect; *F* (9,30) = 341.14, *P* < 0.0001 *η*^2^ = 0.990.

### Pre- and post-treatment effects

All types of stability indices were significantly decreased post-treatment in both groups when compared to values at baseline. In addition, the gross motor record form of BOT-2 and the physical and total scores of PedsQL were significantly increased post-treatment when compared to values at baseline in both groups, Table 3.

**Table 3:** Changes in stability indices, gross motor coordination, and Pedsqol scores between and within groups

	Pre-treatment			Post-treatment				
	Pilates group	Control group		Pilates group	Control group		Pre vs. post Pilates group	Pre vs. post Control group
	Mean ± SD	Mean ± SD	*P* value^a^	Mean ± SD	Mean ± SD	*P* value^a^	*P* value^b^	*P* value^b^
Stability index								
Anteroposterior	1.71 ± 0.26	1.81 ± 0.25	0.280	1.21 ± 0.21	1.67 ± 0.21	0.0001*	0.0001*	0.006*
Mediolateral	2.18 ± 0.19	2.20 ± 0.19	0.683	1.67 ± 0.19	2.06 ± 0.18	0.0001*	0.0001*	0.0001*
Overall	2.63 ± 0.26	2.59 ± 0.25	0.623	2.01 ± 0.24	2.49 ± 0.34	0.0001*	0.0001*	0.001*
Gross motor record form of BOT-2								
Body coordination	27.65 ± 3.03	28.40 ± 2.89	0.428	32.50 ± 2.81	30.01 ± 2.61	0.006*	0.0001*	0.016*
Strength and agility	24.60 ± 2.64	24.85 ± 2.72	0.770	28.45 ± 3.05	26.40 ± 2.71	0.03*	0.0001*	0.0001*
Gross motor composite	25.85 ± 2.81	26.65 ± 2.89	0.381	31.40 ± 3.24	28.95 ± 2.98	0.018*	0.0001*	0.011*
PedsQL								
Physical functioning	65.05 ± 2.28	65.25 ± 2.31	0.789	68.85 ± 2.41	66.40 ± 2.01	0.001*	0.0001*	0.001*
Psychosocial	62.55 ± 2.65	63.70 ± 2.34	0.154	67.81 ± 2.02	64.50 ± 2.72	0.0001*	0.0001*	0.081
Total score	66.85 ± 2.46	66.20 ± 2.88	0.447	70.35 ± 2.76	67.05 ± 2.58	0.0001*	0.0001*	0.011*

*SD* standard deviation, *BOT-2* second edit of Bruininks-Oseretsky of Motor Proficiency, *PedsQL* Pediatric quality of life inventory; *P < 0.05

^a^Comparison between groups

^b^Comparison within group

### Comparison between groups

Between the Pilates and control groups, there were no significant differences in any outcome measures at baseline (*P* > 0.05). All stability indices scores showed a significant decrease and the gross motor record form of BOT-2 and PedsQL scores showed a significant increase in the Pilates group post-treatment when compared to the control group, Table 3.

## Discussion

According to the current study, combining Pilates exercises with a designed physical therapy program resulted in statistically significant improvements in all primary and secondary outcomes when compared to a single application of a designed physical therapy program. According to these results, the study's hypotheses could be rejected.

These results may be related to the increase in muscle strength and endurance of the core muscles of the body after Pilates exercises. According to neurodevelopmental principles, the trunk has an essential role in controlling the movement of the extremities, improving balance, and increasing functional mobility [21]. Training trunk control in addition to gross motor skills has beneficial effects in the treatment of children with movement disorders [22]. Therefore, the significant improvement in balance and gross motor coordination could be attributed to postural control improvement. The findings of a study by Preyal and his colleagues showed that children with DS have poor balance which could be due to the general decrease in muscle tone and muscle strength. They found that trunk muscle strength impacted functional balance from different positions [23] which might illustrate the significant improvements in balance and gross motor coordination that were achieved in the current study.

One factor that might have contributed to the significant difference in the assessed variables in favor of the Pilates group was the pattern of Pilates exercises. Pilates exercises include maintaining a stable posture while concentrating on the respiration rhythm thereby providing a multi-task intervention with increased kinesthetic and proprioceptive awareness and movement co-ordination [24]. Another factor may be the strengthening of deep abdominal muscles through Pilates exercises, which are responsible for core stability and may enhance spinal stability, muscle strength, and pelvic flexibility [7].

Our study findings were congruent with earlier research, which demonstrated that Pilates exercises could improve trunk, lower limbs strength, and balance in children with Cerebral palsy [8–10] and children with juvenile idiopathic arthritis [11].

Children with DS are at increased risk for having problems in their QoL and they are in need of developing interventions aimed at improving QoL in both physical and psychosocial areas [25]. Significant improvement in QoL domains of the Pilates group in our current study in comparison with the control group could be explained by the association between balance and functional abilities in children with motor disabilities [26], as the Pilates exercises allow the child to become more active in daily activities leading to QoL improvement.

The psychosocial aspect of QoL showed significant improvement in the Pilates group only. This could be explained by the fact that Pilates exercises impact the serotonin hormone resulting in reduced depression, relieving tension, improving mood, and heightening concentration, as it actively engages the body and mind [27] which might have a positive influence on the QoL.

Our results were supported by the findings of several studies which showed improvement of QoL after Pilates exercises in children with juvenile idiopathic arthritis [11, 28], adolescents with idiopathic scoliosis [29, 30], female children and adolescents with anorexia nervosa [31], patients with chronic kidney disease [32], patients with multiple sclerosis [33], and subacute stroke patients [12].

To our knowledge, this is the first study assessing the effect of Pilates intervention on balance and gross motor coordination in children with DS. Limited evidence on the effectiveness of Pilates exercises for children with motor difficulties emphasizes the need for additional studies in this area. A recent systematic review and meta-analysis by Cibinello and his colleagues reported that few studies have assessed Pilates intervention impact on children and adolescents [34]. Also, a systematic review by Hornsby and Johnston showed that further research is required to determine the efficacy of Pilates intervention in children with different diagnoses for developing comprehensive guidelines for treatment [35].

This study has several limitations. A small sample size and a specific age range were included. It is recommended to conduct more research using larger sample sizes and diverse age groups. Also, the children's follow-up was not documented. To verify that the exercise effect has persisted, further studies are required.

According to the current study's findings, it could be concluded that adding Pilates exercises to the designed physical therapy program could provide more significant improvements in balance, gross motor coordination, and QoL. So, we recommend adding Pilates exercises to the designed physical therapy program when the aim is to improve these outcomes in children with DS.
